# Supplementary material for: Colour Response in Western Flower Thrips Varies Intraspecifically
Source: Insects. 2022 Jun 10;13(6):538. doi: 10.3390/insects13060538 (PMC9224597; doi:10.3390/insects13060538)
Supplement: Supplementary file 1 [file insects-13-00538-s001.zip › insects-1741607-supplementary.pdf]

Lopez-Reyes et al., Colour Response in Western Flower Thrips varies intraspecifically

Supplementary material

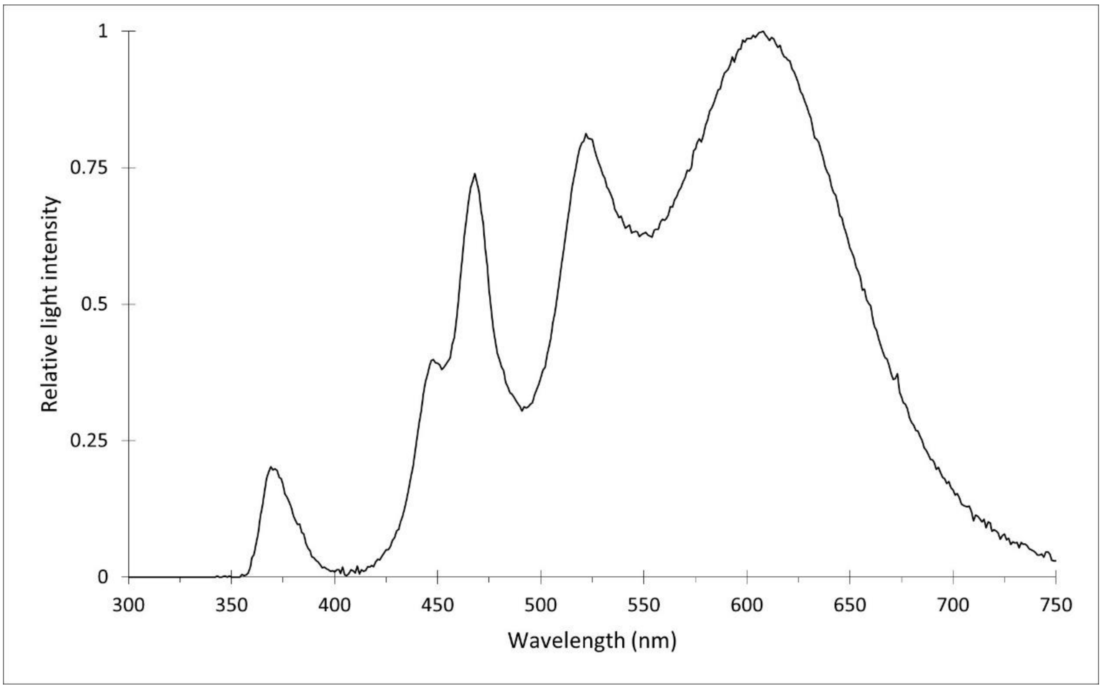

Supplemental Figure S1. Light spectrum of ceiling illumination inside the wind tunnel. The UV-A spectrum (365-390 nm) represents 2% of the amount of light found in the visible light spectrum (400-750 nm).

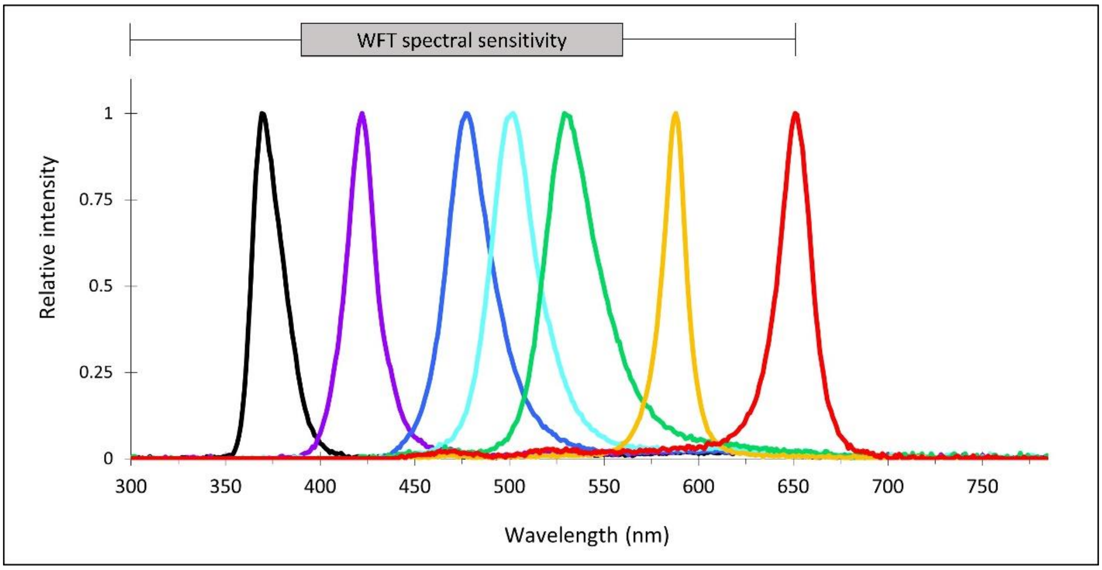

Supplemental Figure S2. Light spectra of LEDs inside the wind tunnel used for Experiment *i*, *ii* and *iii*.

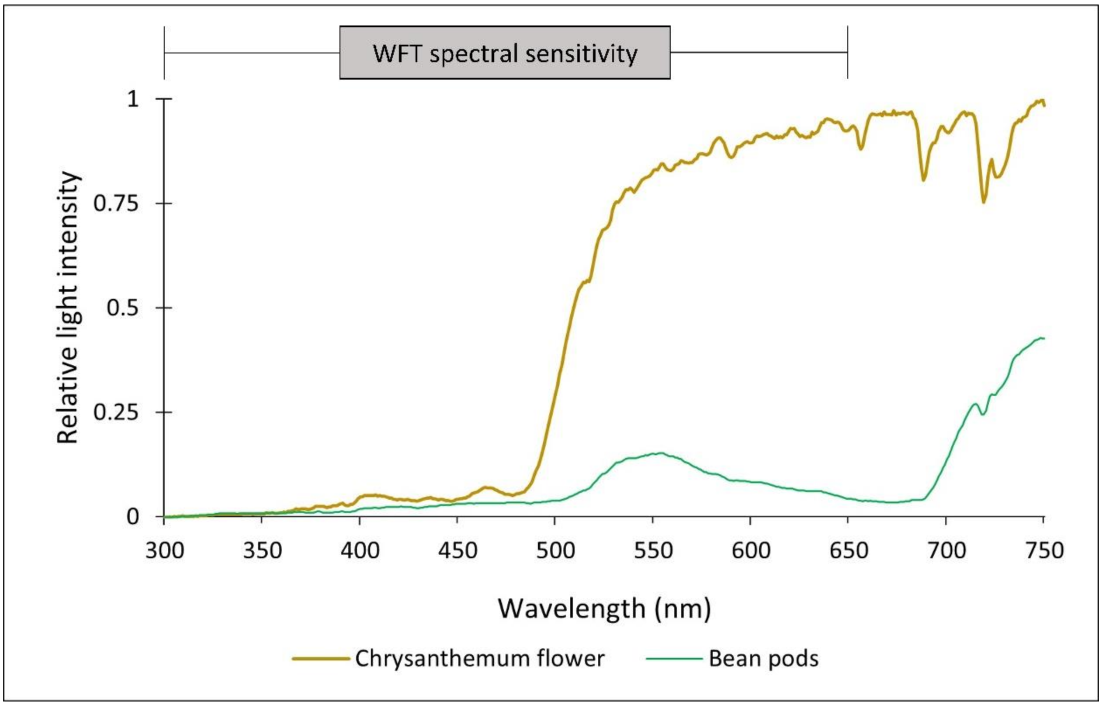

Supplemental Figure S3. Reflectance spectra of chrysanthemum flower and bean pods used for rearing of WFT.

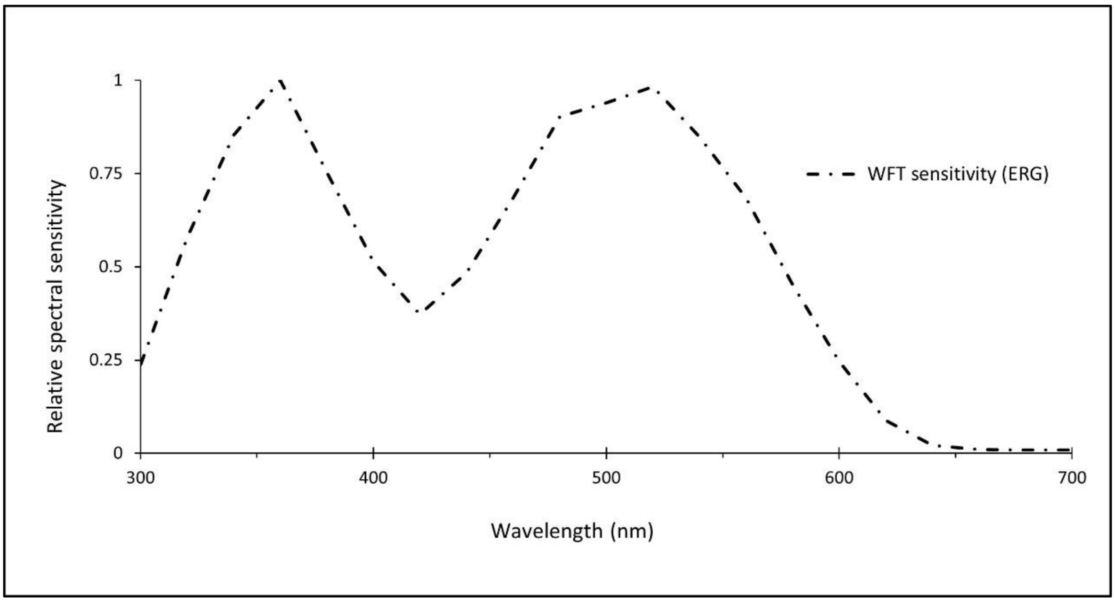

Supplemental Figure S4. Whole eye electroretinogram (ERG) spectral sensitivity curve of western flower thrips (WFT) (*Frankliniella occidentalis*) adapted from Otani et al. [104].

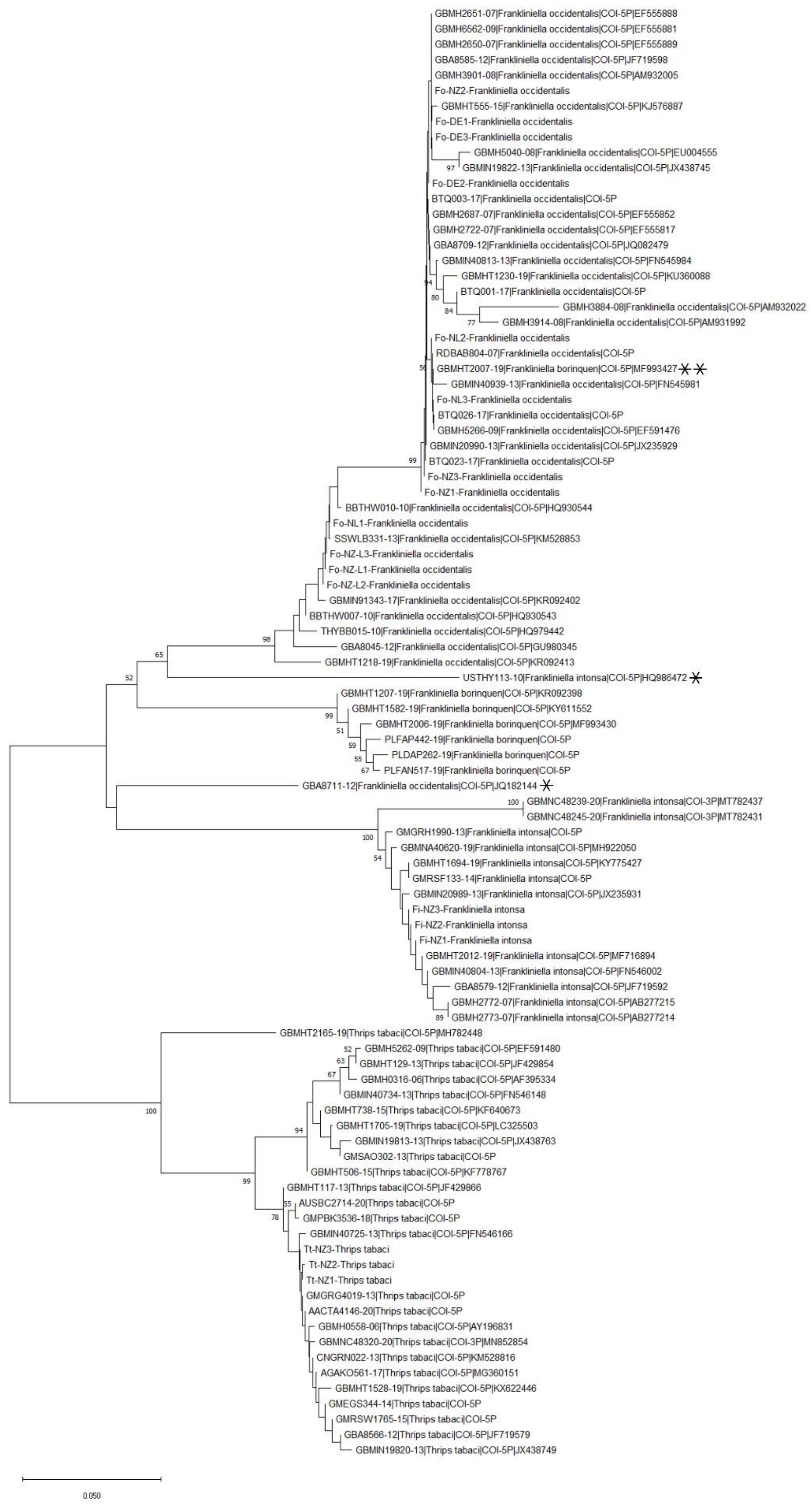

Supplemental Figure S5. COI barcode neighbour-joining tree of sequences generated here for *F. occidentalis* Dutch (Fo-NL1-3) and German (Fo-DE1-3) colonies, New Zealand "glasshouse" (Fo-NZ1-3) and "lupin" strains (Fo-NZ-L1-3), plus New Zealand *F. intonsa* (Fi-NZ1-3) and *Thrips tabaci* (Tt-NZ1-3), together with additional independent Genbank data (**Supplemental Table S3**). Posterior probabilities above 50% are illustrated.

* Reference sequences "GBA8711-12|Frankliniella occidentalis" and "USTHY113-10|Frankliniella intonsa" are considered to be a result of Long Branch Attraction (LBA).

** Reference sequence "GBMHT2007-19|Frankliniella borinquen" is likely to have been misidentified Virgilio et al. [105] in the database based on its position inside the *F. occidentalis* clade and it is not likely to be a result of LBA.

Supplemental Table S1. Specifications of LEDs used (high power 1Watt) for Experiment *i* and *ii***.**

**LED colour**	**Manufacter**	**Model**	**Peak wavelength (nm)**
UV-A	Roithner LaserTechnik GmbH	H2A1-H365-r4	369
Violet	Roithner LaserTechnik GmbH	H2A1-H420	422
Blue	Roithner LaserTechnik GmbH	H2A1-H470	477
Cyan	Roithner LaserTechnik GmbH	H2A1-H490	502
Green	Unknown	Unknown	529
Yellow	Roithner LaserTechnik GmbH	H2A3-H590	588
Red	Roithner LaserTechnik GmbH	H2A1-H650	651

Supplemental Table S2. General information of the 18 specimens used for generating DNA barcode sequences of the thrips used to support species identification and phylogenetic analysis of the Dutch and German *Frankliniella occidentalis* colonies used in this study.

**No.**	**Specimen code and species**	**Country collected**	**Collection date**	**Other information**	**GenBank accession number of COI gene sequence**
**1**	Fo-NZ1- *Frankliniella occidentalis* (NZ)	New Zealand	September, 2020	Laboratory reared colony on chrysanthemum[a]	ON310568
**2**	Fo-NZ2 - *Frankliniella occidentalis* (NZ)	New Zealand	September, 2020	Laboratory reared colony on chrysanthemum[a]	ON310569
**3**	Fo-NZ3 - *Frankliniella occidentalis* (NZ)	New Zealand	September, 2020	Laboratory reared colony on chrysanthemum[a]	ON310570
**4**	Fo-NL1- *Frankliniella occidentalis* (Dutch)	The Netherlands	August, 2020	Laboratory reared NL colony on chrysanthemum[b]	ON310562
**5**	Fo-NL2- *Frankliniella occidentalis* (Dutch)	The Netherlands	August, 2020	Laboratory reared NL colony on chrysanthemum[b]	ON310563
**6**	Fo-NL3- *Frankliniella occidentalis* (Dutch)	The Netherlands	August, 2020	Laboratory reared NL colony on chrysanthemum[b]	ON310564
**7**	Fo-DE1 - *Frankliniella occidentalis* (German)	Germany	March, 2020	Laboratory reared DE colony on bean[c]	ON310565
**8**	Fo-DE2 - *Frankliniella occidentalis* (German)	Germany	March, 2020	Laboratory reared DE colony on bean[c]	ON310566
**9**	Fo-DE3 - *Frankliniella occidentalis* (German)	Germany	March, 2020	Laboratory reared DE colony on bean[c]	ON310567
**10**	Fo-NZ-L1 - *Frankliniella occidentalis* (Lupin)	Rakaia, New Zealand	January, 2019	Collected on yellow tree Lupin	ON310571
**11**	Fo-NZ-L2 - *Frankliniella occidentalis* (Lupin)	Rakaia, New Zealand	January, 2019	Collected on yellow tree Lupin	ON310572
**12**	Fo-NZ-L3 - *Frankliniella occidentalis* (Lupin)	Rakaia, New Zealand	January, 2019	Collected on yellow tree Lupin	ON310573
**13**	Fi-NZ1 - *Frankliniella intonsa*	New Zealand	April, 2021	Laboratory reared colony on bean pods[a]	ON310574
**14**	Fi-NZ2 - *Frankliniella intonsa*	New Zealand	April, 2021	Laboratory reared colony on bean pods[a]	ON310575
**15**	Fi-NZ3 - *Frankliniella intonsa*	New Zealand	April, 2021	Laboratory reared colony on bean pods[a]	ON310576
**16**	Tt-NZ1 - *Thrips tabaci*	New Zealand	April, 2021	Laboratory reared colony on onion[a]	ON310577
**17**	Tt-NZ2 - *Thrips tabaci*	New Zealand	April, 2021	Laboratory reared colony on onion[a]	ON310578
**18**	Tt-NZ3 - *Thrips tabaci*	New Zealand	April, 2021	Laboratory reared colony on onion[a]	ON310579

[a] Plant and Food Research Ltd, Lincoln, Canterbury; [b] Wageningen University and Research; [c] Leibniz University Hannover

Supplemental Table S3. Reference sequences of *Frankliniella occidentalis*, *F. intonsa*, *F. borinquen* and *Thrips tabaci* downloaded from BOLD database for phylogenetic analysis.

No.	Name	Country	BOLD sequence ID	Name
1	GBMHT1207-19|Frankliniella borinquen	Mexico	GBMHT1207-19	GBMHT1207-19|Frankliniella borinquen|COI-5P|KR092398
2	GBMHT1582-19|Frankliniella borinquen	Mexico	GBMHT1582-19	GBMHT1582-19|Frankliniella borinquen|COI-5P|KY611552
3	GBMHT2006-19|Frankliniella borinquen	Mexico	GBMHT2006-19	GBMHT2006-19|Frankliniella borinquen|COI-5P|MF993430
4	GBMHT2007-19|Frankliniella borinquen	Mexico	GBMHT2007-19	GBMHT2007-19|Frankliniella borinquen|COI-5P|MF993427
5	PLDAP262-19|Frankliniella borinquen	Costa Rica	PLDAP262-19	PLDAP262-19|Frankliniella borinquen|COI-5P
6	PLFAN517-19|Frankliniella borinquen	Costa Rica	PLFAN517-19	PLFAN517-19|Frankliniella borinquen|COI-5P
7	PLFAP442-19|Frankliniella borinquen	Costa Rica	PLFAP442-19	PLFAP442-19|Frankliniella borinquen|COI-5P
8	GBA8579-12|Frankliniella intonsa	China	GBA8579-12	GBA8579-12|Frankliniella intonsa|COI-5P|JF719592
9	GBMH2772-07|Frankliniella intonsa	Japan	GBMH2772-07	GBMH2772-07|Frankliniella intonsa|COI-5P|AB277215
10	GBMH2773-07|Frankliniella intonsa	Japan	GBMH2773-07	GBMH2773-07|Frankliniella intonsa|COI-5P|AB277214
11	GBMHT1694-19|Frankliniella intonsa	NA	GBMHT1694-19	GBMHT1694-19|Frankliniella intonsa|COI-5P|KY775427
12	GBMHT2012-19|Frankliniella intonsa	China	GBMHT2012-19	GBMHT2012-19|Frankliniella intonsa|COI-5P|MF716894
13	GBMIN20989-13|Frankliniella intonsa	Serbia	GBMIN20989-13	GBMIN20989-13|Frankliniella intonsa|COI-5P|JX235931
14	GBMIN40804-13|Frankliniella intonsa	United Kingdom	GBMIN40804-13	GBMIN40804-13|Frankliniella intonsa|COI-5P|FN546002
15	GBMNA40620-19|Frankliniella intonsa	NA	GBMNA40620-19	GBMNA40620-19|Frankliniella intonsa|COI-5P|MH922050
16	GBMNC48239-20|Frankliniella intonsa	NA	GBMNC48239-20	GBMNC48239-20|Frankliniella intonsa|COI-3P|MT782437
17	GBMNC48245-20|Frankliniella intonsa	NA	GBMNC48245-20	GBMNC48245-20|Frankliniella intonsa|COI-3P|MT782431
18	GMGRH1990-13|Frankliniella intonsa	Germany	GMGRH1990-13	GMGRH1990-13|Frankliniella intonsa|COI-5P
19	GMRSF133-14|Frankliniella intonsa	Russia	GMRSF133-14	GMRSF133-14|Frankliniella intonsa|COI-5P
20	USTHY113-10|Frankliniella intonsa	USA	USTHY113-10	USTHY113-10|Frankliniella intonsa|COI-5P|HQ986472
21	AACTA4146-20|Thrips tabaci	Australia	AACTA4146-20	AACTA4146-20|Thrips tabaci|COI-5P
22	AGAKO561-17|Thrips tabaci	Canada	AGAKO561-17	AGAKO561-17|Thrips tabaci|COI-5P|MG360151
23	AUSBC2714-20|Thrips tabaci	Australia	AUSBC2714-20	AUSBC2714-20|Thrips tabaci|COI-5P
24	CNGRN022-13|Thrips tabaci	Canada	CNGRN022-13	CNGRN022-13|Thrips tabaci|COI-5P|KM528816
25	GBA8566-12|Thrips tabaci	China	GBA8566-12	GBA8566-12|Thrips tabaci|COI-5P|JF719579
26	GBMH0316-06|Thrips tabaci	NA	GBMH0316-06	GBMH0316-06|Thrips tabaci|COI-5P|AF395334
27	GBMH0558-06|Thrips tabaci	NA	GBMH0558-06	GBMH0558-06|Thrips tabaci|COI-5P|AY196831
28	GBMH5262-09|Thrips tabaci	New Zealand	GBMH5262-09	GBMH5262-09|Thrips tabaci|COI-5P|EF591480
29	GBMHT117-13|Thrips tabaci	USA	GBMHT117-13	GBMHT117-13|Thrips tabaci|COI-5P|JF429866
30	GBMHT129-13|Thrips tabaci	Peru	GBMHT129-13	GBMHT129-13|Thrips tabaci|COI-5P|JF429854
31	GBMHT1528-19|Thrips tabaci	India	GBMHT1528-19	GBMHT1528-19|Thrips tabaci|COI-5P|KX622446
32	GBMHT1705-19|Thrips tabaci	Japan	GBMHT1705-19	GBMHT1705-19|Thrips tabaci|COI-5P|LC325503
33	GBMHT2165-19|Thrips tabaci	Hungary	GBMHT2165-19	GBMHT2165-19|Thrips tabaci|COI-5P|MH782448
34	GBMHT506-15|Thrips tabaci	Kenya	GBMHT506-15	GBMHT506-15|Thrips tabaci|COI-5P|KF778767
35	GBMHT738-15|Thrips tabaci	NA	GBMHT738-15	GBMHT738-15|Thrips tabaci|COI-5P|KF640673
36	GBMIN19813-13|Thrips tabaci	Tanzania	GBMIN19813-13	GBMIN19813-13|Thrips tabaci|COI-5P|JX438763
37	GBMIN19820-13|Thrips tabaci	Madagascar	GBMIN19820-13	GBMIN19820-13|Thrips tabaci|COI-5P|JX438749
38	GBMIN40725-13|Thrips tabaci	United Kingdom	GBMIN40725-13	GBMIN40725-13|Thrips tabaci|COI-5P|FN546166
39	GBMIN40734-13|Thrips tabaci	Israel	GBMIN40734-13	GBMIN40734-13|Thrips tabaci|COI-5P|FN546148
40	GBMNC48320-20|Thrips tabaci	NA	GBMNC48320-20	GBMNC48320-20|Thrips tabaci|COI-3P|MN852854
41	GMEGS344-14|Thrips tabaci	Egypt	GMEGS344-14	GMEGS344-14|Thrips tabaci|COI-5P
42	GMGRG4019-13|Thrips tabaci	Germany	GMGRG4019-13	GMGRG4019-13|Thrips tabaci|COI-5P
43	GMPBK3536-18|Thrips tabaci	Pakistan	GMPBK3536-18	GMPBK3536-18|Thrips tabaci|COI-5P
44	GMRSW1765-15|Thrips tabaci	Russia	GMRSW1765-15	GMRSW1765-15|Thrips tabaci|COI-5P
45	GMSAO302-13|Thrips tabaci	South Africa	GMSAO302-13	GMSAO302-13|Thrips tabaci|COI-5P
46	BBTHW007-10|Frankliniella occidentalis	Canada	BBTHW007-10	BBTHW007-10|Frankliniella occidentalis|COI-5P|HQ930543
47	BBTHW010-10|Frankliniella occidentalis	Canada	BBTHW010-10	BBTHW010-10|Frankliniella occidentalis|COI-5P|HQ930544
48	BTQ001-17|Frankliniella occidentalis	Colombia	BTQ001-17	BTQ001-17|Frankliniella occidentalis|COI-5P
49	BTQ003-17|Frankliniella occidentalis	Colombia	BTQ003-17	BTQ003-17|Frankliniella occidentalis|COI-5P
50	BTQ023-17|Frankliniella occidentalis	The Netherlands	BTQ023-17	BTQ023-17|Frankliniella occidentalis|COI-5P
51	BTQ026-17|Frankliniella occidentalis	The Netherlands	BTQ026-17	BTQ026-17|Frankliniella occidentalis|COI-5P
52	GBA8045-12|Frankliniella occidentalis	NA	GBA8045-12	GBA8045-12|Frankliniella occidentalis|COI-5P|GU980345
53	GBA8585-12|Frankliniella occidentalis	China	GBA8585-12	GBA8585-12|Frankliniella occidentalis|COI-5P|JF719598
54	GBA8709-12|Frankliniella occidentalis	Australia	GBA8709-12	GBA8709-12|Frankliniella occidentalis|COI-5P|JQ082479
55	GBA8711-12|Frankliniella occidentalis	USA	GBA8711-12	GBA8711-12|Frankliniella occidentalis|COI-5P|JQ182144
56	GBMH2650-07|Frankliniella occidentalis	USA	GBMH2650-07	GBMH2650-07|Frankliniella occidentalis|COI-5P|EF555889
57	GBMH2651-07|Frankliniella occidentalis	USA	GBMH2651-07	GBMH2651-07|Frankliniella occidentalis|COI-5P|EF555888
58	GBMH2687-07|Frankliniella occidentalis	New Zealand	GBMH2687-07	GBMH2687-07|Frankliniella occidentalis|COI-5P|EF555852
59	GBMH2722-07|Frankliniella occidentalis	Japan	GBMH2722-07	GBMH2722-07|Frankliniella occidentalis|COI-5P|EF555817
60	GBMH3884-08|Frankliniella occidentalis	South Africa	GBMH3884-08	GBMH3884-08|Frankliniella occidentalis|COI-5P|AM932022
61	GBMH3901-08|Frankliniella occidentalis	Croatia	GBMH3901-08	GBMH3901-08|Frankliniella occidentalis|COI-5P|AM932005
62	GBMH3914-08|Frankliniella occidentalis	Kenya	GBMH3914-08	GBMH3914-08|Frankliniella occidentalis|COI-5P|AM931992
63	GBMH5040-08|Frankliniella occidentalis	Kenya	GBMH5040-08	GBMH5040-08|Frankliniella occidentalis|COI-5P|EU004555
64	GBMH5266-09|Frankliniella occidentalis	Germany	GBMH5266-09	GBMH5266-09|Frankliniella occidentalis|COI-5P|EF591476
65	GBMH6562-09|Frankliniella occidentalis	China	GBMH6562-09	GBMH6562-09|Frankliniella occidentalis|COI-5P|EF555881
66	GBMHT1218-19|Frankliniella occidentalis	Mexico	GBMHT1218-19	GBMHT1218-19|Frankliniella occidentalis|COI-5P|KR092413
67	GBMHT1230-19|Frankliniella occidentalis	India	GBMHT1230-19	GBMHT1230-19|Frankliniella occidentalis|COI-5P|KU360088
68	GBMHT555-15|Frankliniella occidentalis	China	GBMHT555-15	GBMHT555-15|Frankliniella occidentalis|COI-5P|KJ576887
69	GBMIN19822-13|Frankliniella occidentalis	Tanzania	GBMIN19822-13	GBMIN19822-13|Frankliniella occidentalis|COI-5P|JX438745
70	GBMIN20990-13|Frankliniella occidentalis	Serbia	GBMIN20990-13	GBMIN20990-13|Frankliniella occidentalis|COI-5P|JX235929
71	GBMIN40813-13|Frankliniella occidentalis	Italy	GBMIN40813-13	GBMIN40813-13|Frankliniella occidentalis|COI-5P|FN545984
72	GBMIN40939-13|Frankliniella occidentalis	United Kingdom	GBMIN40939-13	GBMIN40939-13|Frankliniella occidentalis|COI-5P|FN545981
73	GBMIN91343-17|Frankliniella occidentalis	Mexico	GBMIN91343-17	GBMIN91343-17|Frankliniella occidentalis|COI-5P|KR092402
74	RDBAB804-07|Frankliniella occidentalis	Canada	RDBAB804-07	RDBAB804-07|Frankliniella occidentalis|COI-5P
75	SSWLB331-13|Frankliniella occidentalis	Canada	SSWLB331-13	SSWLB331-13|Frankliniella occidentalis|COI-5P|KM528853
76	THYBB015-10|Frankliniella occidentalis	USA	THYBB015-10	THYBB015-10|Frankliniella occidentalis|COI-5P|HQ979442

Supplemental Table S4. Results of species identification queries to the BOLD database for the 18 new sequences developed here. BOLD Identification System (IDS) (https://www.boldsystems.org/; accessed 05/03/2021).

Specimen ID	Best ID	Consensus sequence length (bp)	Sequence similarity top match (%)	Sequence similarity low match (%)	Date of ID	Ambiguity
Fo-NZ1	*Frankliniella occidentalis*	594	100	99.75	18/05/2021	(*F. borinquen* possibility)
Fo-NZ2	*Frankliniella occidentalis*	594	100	99.82	18/05/2021	(*F. borinquen* possibility)
Fo-NZ3	*Frankliniella occidentalis*	619	100	99.75	18/05/2021	(*F. borinquen* possibility)
Fo-NL1	*Frankliniella occidentalis*	606	100	99.83	18/05/2021	solid ID
Fo-NL2	*Frankliniella occidentalis*	596	100	99.66	18/05/2021	(*F. borinquen* possibility)
Fo-NL3	*Frankliniella occidentalis*	593	100	99.66	18/05/2021	(*F. borinquen* possibility)
Fo-DE1	*Frankliniella occidentalis*	614	100	99.82	19/05/2021	(*F. borinquen* possibility)
Fo-DE2	*Frankliniella occidentalis*	575	100	99.65	19/05/2021	(*F. borinquen* possibility)
Fo-DE3	*Frankliniella occidentalis*	592	100	99.82	19/05/2021	(*F. borinquen* possibility)
Fo-NZ-L1	*Frankliniella occidentalis*	578	100	99.66	19/05/2021	solid ID
Fo-NZ-L2	*Frankliniella occidentalis*	581	100	99.66	19/05/2021	solid ID
Fo-NZ-L3	*Frankliniella occidentalis*	591	100	99.66	19/05/2021	solid ID
Fi-NZ1	*Frankliniella intonsa*	603	99.83	99.17	18/05/2021	solid ID
Fi-NZ2	*Frankliniella intonsa*	602	99.82	99.29	18/05/2021	solid ID
Fi-NZ3	*Frankliniella intonsa*	590	99.83	98.97	18/05/2021	solid ID
Tt-NZ1	*Thrips tabaci*	637	100	100	19/05/2021	solid ID
Tt-NZ2	*Thrips tabaci*	600	100	100	19/05/2021	solid ID
Tt-NZ3	*Thrips tabaci*	614	100	100	19/05/2021	solid ID

Supplemental Table S5. Mean ± SE estimates of intra- and interspecific genetic distances of thrips COI gene sequences included in the phylogenetic analysis. Distances were calculated using the Kimura 2-parameter model with 1000 bootstrap replications. Intraspecific distances of each group are shown in in bold.

Thrips groups	*Thrips tabaci*	*F. occidentalis* (Lupin)	*F. occidentalis* (German)	*F. intonsa*	*F. occidentalis* (Dutch)	*F. occidentalis*	*F. borinquen*
Thrips tabaci	**2.57 ± 0.4**						
F. occidentalis (Lupin)	26.15 ± 2.6	**0.0**					
F. occidentalis (German)	25.55 ± 2.5	3. 28 ± 0.8	**0.12 ± 0.12**				
F. intonsa	24.64 ± 2.4	22.27 ± 2.2	21.96 ± 2.1	**4.85 ± 0.5**			
F. occidentalis (Dutch)	26.01 ± 2.5	2.31 ± 0.5	1.18 ± 0.3	22.32 ± 2.1	**2.08 ± 0.5**		
F. occidentalis	25.76 ± 2.5	4.09 ± 0.8	1.76 ± 0.2	22.96 ± 2.2	2.63 ± 0.4	**3.61 ± 0.5**	
F. borinquen	23.55 ± 2.3	14.76 ± 1.6	15.28 ± 1.74	21.82 ± 2.1	15.18 ± 1.7	16.05 ± 1.8	**5.72 ± 0.6**

Supplemental Table S6. Summary of results from *Experiment i*: mean percentage of Dutch WFT (NL*c*) caught on sticky plate of the LED lamp at seven different wavelengths as single choice treatments six hours after release (95% confidence limits).

LED Colour	Peak wavelength (nm)	WFT caught (%)
UVA	369	37.3 (28.5 - 46.9)
Violet	422	12.8 (7.6 - 20.6)
Blue	477	21.0 (14.2 - 29.8)
Cyan	502	7.5 (3.8 - 14.4)
Green	529	7.3 (3.6 - 14.1)
Yellow	588	35.5 (26.9 - 45.1)
Red	651	10.8 (6.1 - 18.3)

Supplemental Table S7. Summary of results from *Experiment ii*. Mean percentage of German WFT(DE*c*) attracted to blue and yellow light six hours after release. Comparative data from **Supplemental Table S6** are included for Dutch WFT (NL*c*).

Colony	LED colour	Peak wavelength (nm)	Mean % WFT caught (95% confidence limits), n=4
German	Blue	477	33.8 (25.2, 43.5)
(DEc)	Yellow	588	24.0 (16.7, 33.3)
Dutch	Blue	477	21.0 (14.2, 29.8)
(NLc)	Yellow	588	35.5 (26.9, 45.1)

Supplemental Table S8. Summary of results of *Experiment iii, analysis a*. Percentage of the different host reared WFT colonies on either yellow or blue light of only those that landed on the sticky glass plate of the LED lamp.

Colony	Rearing regime[1]	Mean % WFT (95% confidence limits), n=6	
		Yellow	Blue
German	DE*b*	22.3 (14.4,32.9)	77.7 (67.1,85.6)
	DE*bc*$_{4-8}$	36.5 (27.1,47.1)	63.5 (52.9,72.9)
	DE*bc*$_{12-14}$	39.7 (31.0,49.1)	60.3 (50.9,69.0)
Dutch	NL*b*	50.0 (41.3,58.7)	50.0 (41.3,58.7)
	NL*bc*$_{4-8}$	79.7 (71.2,86.2)	20.3 (13.8,28.8)
	NL*bc*$_{12-14}$	67.2 (59.3,74.1)	32.8 (25.9,40.7)

[1] See Figure 1 in the manuscript

Supplemental Table S9. Summary of results for *Experiment iii*, *analysis b*. Distribution of different host reared WFT colonies at the four locations inside the wind tunnel six hours after release.

Colony	Rearing regime[1]	Mean % WFT (95% confidence limits), n=6			
		Elsewhere	*Ceiling*	*Yellow*	*Blue*
**German**	DE*b*	57.9 (49.2,66.1)	23.4 (16.9,31.5)	4.2 (1.8,9.3)	14.5 (9.4,21.7)
	DE*bc4-8*	54.5 (45.7,63.0)	23.9 (17.2,32.1)	7.9 (4.3,14.1)	13.7 (8.7,20.9)
	DE*bc12-14*	50.6 (41.9,59.1)	22.6 (16.2,30.7)	10.7 (6.4,17.3)	16.2 (10.8,23.6)
**Dutch**	NL*b*	65.2 (56.5,73.1)	3.9 (1.6,9.2)	15.4 (10.1,22.9)	15.4 (10.1,22.8)
	NL*bc4-8*	45.8 (37.3,54.6)	26.5 (19.5,34.9)	22.1 (15.7,30.3)	5.6 (2.7,11.3)
	NL*bc12-14*	45.3 (36.9,54.0)	17.2 (11.6,24.8)	25.1 (18.4,33.4)	12.3 (7.6,19.2)

[1] See Figure 1 in the manuscript

References (Supplementary materials)

104. Otani, Y.; Wakakuwa, M.; Arikawa, K. Relationship between Action Spectrum and Spectral Sensitivity of Compound Eyes Relating Phototactic Behavior of the Western Flower Thrips, *Frankliniella Occidentalis*. *Jpn. J. Appl. Entomol. Zool.* **2014**, *58*, 177–185, doi:10/gn27z4.

105. Virgilio, M.; Backeljau, T.; Nevado, B.; De Meyer, M. Comparative Performances of DNA Barcoding across Insect Orders. *BMC Bioinformatics* **2010**, *11*, 1–10, doi:10/ch2bd9.
